# The Impact of the CB_2_ Cannabinoid Receptor in Inflammatory Diseases: An Update

**DOI:** 10.3390/molecules29143381

**Published:** 2024-07-18

**Authors:** Volatiana Rakotoarivelo, Thomas Z. Mayer, Mélissa Simard, Nicolas Flamand, Vincenzo Di Marzo

**Affiliations:** 1 Centre de Recherche de l’Institut Universitaire De Cardiologie Et De Pneumologie de Québec, Département of Médecine, Université Laval, Québec City, QC G1V 4G5, Canada; 2 Canada Excellence Research Chair on the Microbiome-Endocannabinoidome Axis in Metabolic Health (CERC-MEND), Université Laval, Québec City, QC G1V 0V6, Canada; 3 Institut sur la Nutrition et les Aliments Fonctionnels, and Centre NUTRISS, École de Nutrition, Université Laval, Québec City, QC G1V 0V6, Canada; 4 Joint International Unit between the CNR of Italy and Université Laval on Chemical and Biomolecular Research on the Microbiome and Its Impact on Metabolic Health and Nutrition (UMI-MicroMeNu), Québec City, QC G1V 0V6, Canada

**Keywords:** inflammatory diseases, inflammation, cannabinoid, endocannabinoid, cannabinoid receptor

## Abstract

The emergence of inflammatory diseases is a heavy burden on modern societies. Cannabis has been used for several millennia to treat inflammatory disorders such as rheumatism or gout. Since the characterization of cannabinoid receptors, CB_1_ and CB_2_, the potential of cannabinoid pharmacotherapy in inflammatory conditions has received great interest. Several studies have identified the importance of these receptors in immune cell migration and in the production of inflammatory mediators. As the presence of the CB_2_ receptor was documented to be more predominant in immune cells, several pharmacological agonists and antagonists have been designed to treat inflammation. To better define the potential of the CB_2_ receptor, three online databases, PubMed, Google Scholar and clinicaltrial.gov, were searched without language restriction. The full texts of articles presenting data on the endocannabinoid system, the CB_2_ receptor and its role in modulating inflammation in vitro, in animal models and in the context of clinical trials were reviewed. Finally, we discuss the clinical potential of the latest cannabinoid-based therapies in inflammatory diseases.

## 1. Introduction

Inflammatory diseases are the leading causes of disability and death throughout developing countries [1]. Therefore, it is more and more urgent to find a therapeutic target that could improve the conditions of people suffering from inflammatory diseases [2]. These disorders evolve from a sustained activation of the immune system that can be localized and/or disseminated throughout the body [3]. Chinese traditional healers have used cannabis to treat inflammation since 2000 before Common Era [4]. Since the identification of cannabinoid receptors in immune cells, the potential of cannabinoid pharmacotherapy in pain and inflammatory conditions has received much attention [5]. Here, we are updating the literature review by Turcotte et al. [6], highlighting the studies of the last decade on the role of the CB_2_ receptor in peripheral inflammation and its potential as a pharmaceutical target in inflammatory diseases.

## 2. The Type 2 Cannabinoid Receptor (CB_2_)

Our knowledge of the endocannabinoid system started with the elucidation of the structure, metabolism and function of Δ^9^-tetrahydrocannabinol (Δ^9^-THC) [7,8,9]. The first cannabinoid receptor CB_1_ was isolated and cloned from a rat cerebral cortex cDNA library. The translated sequence of the identified cDNA was a 473 amino acid protein sequence and a member of the G-protein-coupled family of receptors [10]. Two years later, the human cannabinoid receptor CB_2_ was cloned from the promyeloid cell line HL-60 [11]. The protein encoded by a sequence of 360 amino acids was found to have 44% homology with the CB_1_ receptor [12,13]. The CB_2_ receptor is considered a peripheral receptor due to its predominant expression in various immune cells [14,15]. Eosinophils, B cells and natural killer (NK) cells were found to strongly express CB_2_, whereas monocytes, neutrophils, helper T cells and cytotoxic T cells showed mRNA transcript levels ranging from moderate to low [16,17]. The membrane expression of CB_2_ was also accessed by flow cytometry, and it was shown that circulating B cells, NK cells and monocytes express higher levels of the receptor compared to CD4^+^T lymphocytes, CD8^+^T lymphocytes and neutrophils [18]. Another study confirmed the membrane expression of CB_2_ on eosinophils and found that it was increased in eosinophils isolated from the blood of allergic patients compared to those isolated from healthy donors [19]. Furthermore, functional CB_2_ is detected in perivascular microglia [20] and the brain [21]. Therefore, it is suggested that CB_2_ is also expressed in the healthy central nervous system albeit at lower transcript levels and not necessarily in neurons [21].

To better understand the expression of CB_2_ in the inflammatory context, Turcotte et al. [6] and others comprehensively reviewed the expression of CB_2_ in human tissues and animal models and highlighted the role of CB_2_ as a regulator of inflammation. For instance, in the gastrointestinal tract, CB_2_ is detected in the esophagus, stomach and ileum [22]. In humans, CB_2_ is expressed by colonic epithelial cells but only under inflammatory conditions [23]. The expression of CB_2_ by the liver is specifically high during embryonic development [24], and its expression by hepatic myofibroblasts increases under pathological conditions such as liver fibrosis [25] and liver injury [26]. In the cardiovascular system, CB_2_ is expressed by cardiomyocytes and endothelial cells [27], and its expression increases in an inflammatory context such as atherosclerosis and acute myocardial infarction [28]. In the musculoskeletal system, the protein expression of CB_2_ by chondrocytes has been detected and shown to be increased in the joint tissues of rats [29,30] and humans with osteoarthritis [31]. In the brain, CB_2_ expression is still controversial, as it seems to be detected only in the context of neuroinflammation and is attributed to microglia [32]. However, other studies have reported that CB_2_ is detected in neurons and may mediate brain functions [33]. Moreover, the expression of this receptor in innate and adaptive immune cells was recently reviewed by Simard and colleagues [17]. In particular, they showed that high CB_2_ (*Cnr2*) expression is found in eosinophils and B cells, while low expression is found in T cells and monocytes [17]. Additionally, the expression of CB_2_ by different tissues is upregulated in an inflammatory context including neuroinflammation [34,35,36] and acute and chronic inflammation [37,38]. Considering the relevance of the CB_2_ receptor in the regulation of the immune response, CB_2_ is seen as a potential therapeutic target in inflammatory diseases.

## 3. CB_2_ Signaling Pathway

**Figure 1:**
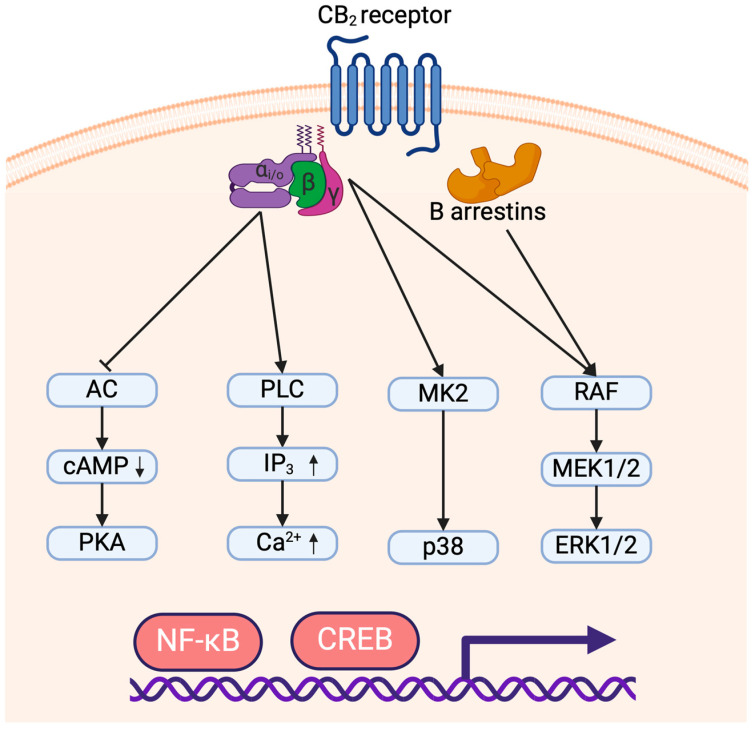
Signaling of the CB_2_ receptor leading to pro- and anti-inflammatory responses. The arrows (↑/↓) indicate an increase or decrease in molecule concentration, respectively.

CB_1_ and CB_2_ are both G-protein-coupled receptors binding to a trimer of Gα/β/γ proteins and activate in part similar signaling cascades. CB_1_ can couple to either Gαs or Gαi/o proteins which respectively activate or inhibit the adenylyl cyclases. Conversely, CB_2_ almost exclusively recruits Gαi/o proteins leading to inhibition of adenylyl cyclase activity which reduces AMPc levels and prevents activation of PKA [39]. Since PKA activation leads to NF-κB and CREB transactivation [40], Gi/o signaling triggered by CB_2_ is believed to prevent the induction of inflammatory genes. Although CB_2_ coupling to Gαs has been reported only in rare instances, it was suggested to play an important role in the induction of a pro-inflammatory response dependent on PKA activation [41]. CB_2_ engagement also leads to the Gβ- and Gγ-dependent activation of p38 and ERK1/2 MAP kinases [41,42]. CB_2_-dependent MAPK phosphorylation was shown to be responsible for the activation of pro-inflammatory genes through transcription factors NF-κB and CREB [41,43]. Moreover, contrarily to CB_1_, CB_2_ cannot trigger potassium channel signaling through Gαs but can initiate Ca^2+^ signaling via phospholipase C activation by Gαi/o proteins [44]. Finally, the c-terminal tail of CB_1_ and CB_2_ can also be phosphorylated by G-Protein-Coupled Receptor Kinases (GRK), allowing the recruitment of β-arrestins. CB_2_ can bind β-arrestin1 and β-arrestin2 which leads to receptor internalization but also to the phosphorylation of MAPK ERK1/2 [45,46] (Figure 1).

A single GPCR can adopt different conformations depending on its interaction with specific agonists, and some conformations can trigger only part of the signaling events attributed to the receptor [47]. Evidence of this mechanism, termed biased agonism, has been reported for both CB_1_ and CB_2_. In the case of CB_2_, THC strongly potentiates ERK phosphorylation but is less effective on β-arrestin recruitment and cAMP signaling and does not activate G-protein-coupled inwardly rectifying potassium channels (GIRK). Endocannabinoids can activate all signaling pathways, but *N*-arachidonoylethanolamine (AEA) strongly increases GIRK and ERK activation and is less impactful on cAMP and β-arrestin signaling, while 2-AG favors GIRK signaling to other pathways. As for selective CB_2_ agonists, JWH-133 induces β-arrestin and cAMP signaling more potently than GIRK and ERK activation, while HU910 and HU308 do not show a strong bias toward a specific signaling pathway [43,48]. Given that CB_2_ agonists vary greatly in their selectivity, efficacy and capacity to trigger signaling events, studies using different agonists must be compared carefully, and agonists that are both selective and not greatly biased toward signaling pathways should be favored in future studies.

Adenylyl cyclase (AC); cAMP Response Element-Binding Protein (CREB); Cyclic adenosine monophosphate (cAMP); Extracellular signal-regulated kinase (ERK); Inositol trisphosphate (IP3); Mitogen-activated protein kinase kinase (MEK); Mitogen-activated protein kinase-activated protein kinase-2 (MK2); Nuclear factor kappa-light-chain-enhancer of activated B cells (NF-κB); p38 mitogen-activated protein kinase (p38); Phospholipase C (PLC); Protein kinase A (PKA); Protein kinase C (PKC); Rapidly accelerated fibrosarcoma kinase (RAF).

## 4. *Cnr2*-KO Mice and Inflammation

*Cnr2*^c^ deficient animals provide a powerful tool to understand the roles of CB_2_ in inflammatory mouse models [49]. A majority of studies showed that *Cnr2*-deficient mice have a pro-inflammatory profile (Table 1), implying that CB_2_ is involved in the negative regulation of the inflammatory response [50,51,52,53,54,55]. In inflammatory models such as lipopolysaccharide (LPS)-induced inflammation [54,56] or alcoholic liver disease [50], the genetic deletion of *Cnr2* was associated with an increased production of pro-inflammatory cytokines. The secretion of chemokines and the expression of their receptors also increased leading to a modification of cell migration and infiltration. In acute inflammatory conditions, such as the murine dorsal air pouch model, the absence of *Cnr2* altered the recruitment of neutrophils to the site of inflammation [54].

On the other hand, some studies have shown that *Cnr2* deficiency can also dampen inflammation, notably, inflammatory states involving eosinophils, as we summarize in Table 2. For instance, mouse models of allergic bronchitis are characterized by the infiltration of activated eosinophils in the airways [57]. This could be explained, in part, by the ability of 2-AG, concomitantly with IL-5, to act as a chemoattractant for eosinophils [58]. Eosinophil accumulation often correlates positively with disease severity and immune cell infiltration and is effectively reversed by genetic deletion of *Cnr2* [59]. The absence of *Cnr2* alters innate immune cell function as it reduces the ability of dendritic cells to express costimulatory molecules that affect T cell activation and proliferation [53]. Furthermore, CB_2_ signaling affects macrophage functions by steering them toward those typical of an M2 phenotype [60].

These contrasting studies demonstrate that the inflammatory effect of *Cnr2* deletion is not completely understood. It is also crucial to take into consideration other receptors and endocannabinoid-like compounds belonging to the endocannabinoidome (see below), which can compensate for the absence of CB_2_ (Table 1 and Table 2) [61,62,63]. Among these receptors, we highlight PPARγ (peroxisome proliferator-activator receptor gamma), which belongs to the endocannabinoidome [64]. Through its affinity for endocannabinoids, PPARγ can downregulate the activation, proliferation and migration of helper T cells [65,66,67,68]. Considering that the endocannabinoid system is involved in key biological processes, effects seen in *Cnr2*^−^*^/^*^−^ inflammation mice models cannot be strictly attributed to a direct implication of CB_2_ in inflammation. Endocannabinoids can also act through other receptors, namely transient receptor potential vanilloid type 1(TRPV1) [69] and PPARs [70], which can counteract the absence of CB_2_ and explain the variable outcomes in response to the genetic deletion of *Cnr2* [64].

**Table 1:** Pro-inflammatory effects of *Cnr2* genetic deletion in mouse models of inflammation.

Model	Species	Effects	References
Alcoholic liver disease	C57BL/6N	↑ CCL3 ↑ TNF-α, IL-6, IL-1β, IL-1α	[50]
Concavalin A-induced acute liver injury	C57BL/6J	↑ Liver injury ↑ Macrophage proliferation and activation ↑ TNF-α	[51]
Corneal injury	BALB/c	↑ Neutrophil infiltration	[52]
DNFB-induced hypersensitivity	C57BL/6J	↑ Neutrophil recruitment ↑ Ear swelling ↓ CCR7 and CXCR4 ↓ MHC II and CD40/CD86 expression by dendritic cells	[53]
Dorsal air pouch model	C57BL/6J and B6.SJL	↑ Neutrophil migration ↑ MMP-9, CCL2, CCL4, CXCL1, CXCL2, CXCL5, CXCL10 ↑ IL-6	[54]
Endotoxin-induced uveitis	BALB/c	↑ Endothelial leukocytes adhesion ↑ Neutrophil migration	[71]
Excisional skin wound	C57BL/6	↑ IL-6 and TNF-α	[72]
Hepatic ischemia-reperfusion injury	C57BL/6	↑ Neutrophil recruitment ↑ Inflammatory cytokines ↑ Liver damage	[73]
LPS-induced inflammation	C57BL/6J	↑ Neutrophil recruitment to the spleen ↑ Leukocyte mobilization ↑ MMP-9 ↑ CCL2, CXCL10, ↑ IL-6	[54,56,74]
Myocardial ischemia-reperfusion injury	C57BL/6J	↑ Neutrophil and macrophage infiltration ↓ IL-10	[75]
Obesity	C57BL/6J	↑ Obesity ↑ Macrophage infiltration in adipose tissue ↑ CCL2 ↑ TNF-α	[55,76]
TNBS-induced colitis	C57BL/6	↑ Colitis ↑ TNF-α and IL-1β	[77]
Traumatic brain injury	C57BL/6	↑ TNF-α, iNOS and ICAM mRNA ↑ Blood–brain barrier permeability	[78,79]

The arrows (↑/↓) indicate whether a process is enhanced or reduced, respectively.

**Table 2:** Anti-inflammatory effects of *Cnr2* genetic deletion in mouse models of inflammation.

Model	Species	Effects	References
Cecal-ligation-induced sepsis	C57BL/6J	↓ Bacterial invasion ↓ Mortality ↓ IL-10	[80]
DSS-induced colitis	C57BL/6J	↑ Expansion of regulatory CX3CR1^hi^ macrophages (M_2_ macrophages) ↓ NLRP3 inflammasome activation in macrophages	[81]
Obesity	C57BL/6J	↑ Insulin sensitivity ↓ CCL2 ↓ TNF-α, IL-6 and CCL2	[82]
Plasmodium falciparum infection (Malaria)	C57BL/6J	↓ CCL17 ↓ IFN-γ and TNF-α	[83]
Traumatic brain injury	C57BL/6	↓ Neurological deficits ↓ Edema and blood–brain barrier permeability	[78,79]
Type-1 diabetes	C57BL/6 and NOD/Lt	↑ CX3CR1^hi^ macrophages	[60]

The arrows (↑/↓) indicate whether a process is enhanced or reduced, respectively.

### 4.1. Endogenous Ligands

Endogenous molecules with similar functions as Δ^9^-THC were discovered and named “endocannabinoids” [84]. To date, there are two characterized endocannabinoids binding to the cannabinoid receptors [7,13,85,86]. *N*-arachidonoylethanolamine (AEA), also known as anandamide, was first isolated from the porcine brain [7]. The second molecule, 2-arachidonoylglycerol (2-AG), was next found in canine intestinal tissue [86]. 2-AG has a higher efficacy at CB_2_ than AEA [64,85]. Moreover, since AEA is a partial agonist at CB_2_ as compared to 2-AG, when both ligands are present in a competitive way, AEA may act as a competitive antagonist of CB_2_ in the presence of 2-AG [87].

Other candidate endocannabinoids derived from arachidonic acid have also been identified. The ester form of arachidonic acid coupled to glycerol, also known as Noladin or 2-AGE, was isolated from the porcine brain in 2001 by Hanus et al. [88]. Based on the selective affinity of Noladin for the CB_1_ receptor [89], it took longer to identify that Noladin can also act as a full agonist at the CB_2_ receptor [90,91]. However, in areas such as the brain, where endocannabinoids and their receptors are known to be abundant, the concentration of 2-AGE was similar to that of AEA. [92]. In addition, Oka and colleagues showed that in mammals, the ratio of 2-AGE to 2-AG was very low, ranging from 1/500 to 1/4000 [93]. Although 2-AGE can act via the two endocannabinoid receptors, the low presence of Noladin both centrally and peripherally could raise the question of the physiological relevance of Noladin. The endocannabinoid family also includes O-arachidonoyl-ethanolamine, also known as Virodhamine, which is the ester of arachidonic acid and ethanolamine [94]. Discovered in 2002 by Porter et al., Virodhamine is known to be a CB_2_ agonist [94]. In peripheral tissues where CB_2_ is expressed, such as the spleen, Virodhamine levels were eight times higher than AEA. Although Virodhamine is known to be a CB_2_ agonist, it has been shown that it is also an agonist of the GPR55 receptor and that it exerts its effects through this receptor [95]. The third component results from the conjugation of arachidonic acid with dopamine, *N*-arachidonoyl-dopanime, also known as NADA [96], and has been reported to be present in specific regions of the brain including the striatum and hippocampus [96,97]. NADA is an endogenous ligand for CB_1_ but also for transient receptor potential vanilloid type 1 (TRPV1) and exerts its role in the inflammatory response through this receptor [98]. Given that these endocannabinoid ligands activate other receptors of the endocannabinoid system and the endocannabinoidome, such as CB_1_, GPR55 and TRPV1 [96], in this review, we will focus on endogenous ligands that exert an effect through the CB_2_ receptor.

Both AEA and 2-AG are lipid mediators derived from the cleavage of membrane phospholipid precursors [99]. However, their biosynthesis depends on different enzymatic pathways. Amongst the four known pathways leading to the production of AEA, the most studied is the hydrolysis of *N*-arachidonoyl-phosphatidylethanolamine (NAPE) by the *N*-acyl-phosphatidylethanolamine phospholipase D (NAPE-PLD) [100]. AEA is primarily degraded by fatty acid amide hydrolase 1 (FAAH_1_), a serine hydrolase active at alkaline pH [101]. *N*-acylethanolamine-selective acid amidase (NAAA), which is a cysteine hydrolase working in an acidic environment, is another important catabolic enzyme degrading AEA, although with lower affinity/efficacy than with other *N*-acyl-ethanolamines, such as *N*-palmitoyl-ethanolamine. Indeed, with varying degrees of selectivity, these enzymes can hydrolyze N-acylethanolamines to fatty acids and ethanolamine [102].

As for 2-AG, it notably arises from the conversion of a phospholipid into 1/2-diacylglycerol (DAG), followed by the hydrolysis of the latter by the DAG lipases (DAGL) α or β [103], but other biosynthetic pathways have also been documented [104]. Like its 2-monoacylglycerol congeners, 2-AG is mainly catabolized by the monoacylglycerol lipase (MAGL) [105]. AEA, 2-AG, their *N*-acylethanolamine and 2-monoacylglycerol congeners and other long-chain fatty acid amides and their receptors constitute the endocannabinoidome [64].

In an attempt to manipulate the bioavailability of endocannabinoids and enhance their benefits on the immune system response, several inhibitors of FAAH and MAGL were developed [101,106]. Namely, selective inhibition of FAAH with URB597 is associated with increased levels of *N*-acylethanolamines such as AEA and reduced production of pro-inflammatory cytokines and immune cell infiltration in the ovalbumin-induced allergic asthma model [107]. A study by Genovese et al., confirmed that inhibition of FAAH activity by URB878 significantly reduced the inflammation associated with acute lung injury [108]. The genetic deletion of *Mgll*, which encodes the MAGL, is linked to increased levels of monoacylglycerols [109] and protects mice from diet-induced obesity and associated inflammatory diseases [110]. Furthermore, the absence of *Mgll* helps to reduce inflammation and liver damage when mice are exposed to carbon tetrachloride [111]. In addition, using JZL184 to inhibit the activity of MAGL produces antiarthritic effects and reduces the paw inflammation associated with collagen-induced arthritis [112]. These results suggest that endocannabinoids modulate inflammation, although these effects cannot be necessarily attributed to CB_2_ activation.

The activation of the CB_2_ receptor by endogenous ligands was specifically examined in inflammatory models as detailed in Table 3. Briefly, treatments that target CB_2_ either with Δ^9^-THC [113] or the two endocannabinoids AEA and 2-AG [6,114] revealed a dichotomy regarding inflammatory responses. First, most studies highlighted a reduction in immune cell infiltration and secretion of inflammatory mediators associated with CB_2_ activation [115,116,117,118]. More importantly, these treatments are accompanied by an improvement in symptoms associated with inflammation. For example, Madig et al. observed reduced edema and improved blood–brain barrier function and neurological recovery after treatment with 2-AG in order to specifically target the CB_2_ receptor in Theiler’s murine encephalomyelitis virus-induced demyelination [79]. Conversely, other authors have shown that a pro-inflammatory response is associated with cannabinoid treatment as well as selective CB_2_ receptor agonists. As described by Oka et al. in mice treated with oxazolone to induce dermatitis, the number of infiltrating eosinophils is reduced when CB_2_ is blocked with SR144528 [119]. However, the pro-inflammatory effects of endocannabinoids in some cases can be attributed to the products of endocannabinoid metabolism [114,120]. Eicosanoid metabolites derived from arachidonic acid oxidation contribute to inflammation (see Dennis et al. for details [121]). In addition, arachidonic acid and its metabolites modulate type 2 immune responses, which are important in the allergic response through actions on eosinophils and neutrophils [122].

Though the large body of evidence supporting endocannabinoids as anti-inflammatory mediators is cohesive and well established, these contradictory results reaffirm the need to investigate further the mechanisms by which endocannabinoids can exert pro-inflammatory effects.

**Table 3:** Effects of endocannabinoid treatment in rodent models of inflammation.

Model	Species	Treatment	CB_2_-Dependent Validation	Application	Effects	References
Anti-inflammatory effects						
Atherosclerosis	C57BL/6 NOD/SCID DBA/1J C57BL/6 ABH C57BL/6J C57BL/6 BALB/c C57BL/6 C57BL/6 C57BL/6J C57BL/6 C57BL/6J C57BL/6J C57BL/6	Δ^9^-THC 0.1 to 10 mg/kg	SR144528 0.7 mg/kg	Oral administration	↓ Atherosclerotic lesion ↓ Macrophage infiltration ↓ Leukocyte adhesion	[115]
		ApoE/MGL-DKO	SR144528 0.01 mg/kg	Oral administration	↓ Atherosclerotic lesion ↓ Leukocyte infiltration	[109]
Collagen-induced arthritis		JZL184 8 and 40 mg/kg	SR144528 3 mg/kg	Subcutaneous injection	↓ Paw inflammation	[112]
Experimental autoimmune encephalomyelitis		Δ^9^-THC 2.5 to 25 mg/kg	*Cnr2^-/-^*	Intraperitoneal injection	↓ Monocyte recruitment ↓ IFN-γ and IL-2 ↓ T cell proliferation	[116]
Hepatic ischemia-reperfusion injury		HU910 3 and 10 mg/kg	SR144528 3 mg/kg	Intraperitoneal injection	↓ Hepatic injury ↓ CCL3, CXCL2 and TNF-α ↓ Neutrophil infiltration	[117]
Influenza virus infection		Δ^9^-THC 75 mg/kg	*Cnr2* ^−/−^	Oral administration	↓ Lymphocyte and monocyte recruitment ↓ Viral hemagglutinin	[118]
*L. pneumophila* infection		Δ^9^-THC 8 mg/kg	*Cnr2* ^−/−^	Intravenous injection	↓ IFN-γ and IL-12	[123]
ConA-induced hepatitis		AEA 20 mg/kg	SR144528 10 to 20 mg/kg	Intraperitoneal injection	↓ Inflammatory cytokines	[73]
Theiler’s murine encephalomyelitis virus-induced demyelination disease		HU914 5 and 10 mg/kg	*Cnr2* ^−/−^	Intraperitoneal injection	↓ Neurological deficits ↓ Edema and blood–brain barrier permeability	[79]
Carrageenan-induced acute inflammation		URB602 10 and 20 mg/kg	SR144528 1 mg/kg	Intraperitoneal injection	↓ Edema ↓ Nociception	[124]
Cecal-ligation- and puncture-induced sepsis		HU308 2.5 mg/kg	AM630 2.5 mg/kg	Intraperitoneal injection	↓ Inflammatory cytokines ↓ Pyroptosis and NLRP3 activity	[125]
Experimental autoimmune encephalomyelitis		WWL70 5 mg/kg	AM630 3 mg/kg	Intraperitoneal injection	↓ iNOS, COX-2, TNF-α and IL-1β ↓ T cell infiltration ↓ Microglial activation ↓ NF-κB activation	[126]
LPS-induced tactile allodynia		URB597 1 and 10 mg/kg	*Cnr2* ^−/−^	Intraperitoneal injection	↓ Leukocyte rolling ↓ Microvascular perfusion	[74]
LPS-induced acute lung injury		JZL184 16 mg/kg	AM630 2.5 and 5 mg/kg	Intraperitoneal injection	↓ Leukocyte infiltration ↓ BALF cytokines and chemokines	[127]
LPS-induced inflammatory pain	C57BL/6J C57BL/6J	*Faah* ^−/−^	SR144528 3 mg/kg	Intraperitoneal injection	↓ Edema ↓ TNF-α and IL-1β	[128]
		*Faah* ^−/−^	*Cnr2* ^−/−^	-	↓ Allodynia	[74]
Pro-inflammatory effects						
Type-1 diabetes	NOD ICR ICR C57BL/6	AEA 250 μg to 500 μg/ 100ul	*Cnr2* ^−/−^	Oral administration	↓ CX3CR1^+^ macrophages	[60]
Oxazolone-induced dermatitis		2-AG 30 μg dissolved in acetone	SR144528 30 μg dissolved in acetone	Topical application	↑ Eosinophil recruitment ↑ Swelling ↑ CCL2, CCL3 and TNF-α	[129]
TPA-induced ear inflammation		2-AG 30 μg dissolved in acetone	SR144528 30 μg dissolved in acetone	Topical application	↑ Neutrophil recruitment ↑ Swelling ↑ LTB_4_ synthesis	[119]
Pancreatic cancer		2-AG 20 mg/kg	AM630 (ND)	Intraperitoneal injection	↓ Cancer cells proliferation ↓ Dendritic cell activation	[130]

The arrows (↑/↓) indicate whether a process is enhanced or reduced, respectively.

### 4.2. Synthetic Ligands

Studies on marijuana users prompted the potential therapeutic importance of exogenous, synthetic CB_2_ receptor agonists even before the identification of endocannabinoid ligands and receptors and inhibitors of endocannabinoid-inactivating enzymes.

In a multivariate study by Tindall et al., conducted on human immunodeficiency virus (HIV)-positive patients who had not yet developed acquired immunodeficiency syndrome, marijuana users were more likely to have a lower percentage of CD4+ cells and a higher percentage of CD8+ cells [131]. In addition, HIV carriers who use marijuana exhibit an increased risk of developing bacterial pneumonia and other opportunistic infections [132]. Indeed, it was demonstrated that alveolar macrophages in the lungs of marijuana smokers were less effective in their ability to clear bacteria and tumor cells [133]. These studies suggest that natural cannabinoids capable of activating CB_2_ receptors affect the immune system, and this effect could be exploited as a treatment.

Synthetic compounds such as CP55,940 and WIN55,212-2 were available when CB_2_ was cloned, although these synthetic cannabinoids can also activate CB_1_ with similar efficiency [11,134]. A highly selective antagonist for CB_2_ receptors, SR144528, was the first molecule designed in order to investigate CB_2_-mediated endocannabinoid functions in the immune system [135]. Since then, researchers have developed a wide variety of CB_2_-specific antagonists or agonists (Table 4) [136].

In 2017, Soethoudt et al. tested 18 pharmacological agonists and antagonists used in preclinical models and concluded that JWH-133, HU308 and HU910 are the agonists with the best affinity for CB_2_ and least psycho-chemical consequence [48]. JWH-133 (*Ki* = 3.4 nM) was characterized in 1999 and found to be a potent CB_2_ agonist, 200 times more selective for CB_2_ than for CB_1_ [13,137]. HU308 (*Ki* = 22.7 nM) is a specific agonist for CB_2_ and does not appreciably bind to CB_1._ Of these three specific agonists, HU910 (*Ki* = 6 nM) is one of the most recently developed as it was first used in 2012 and has specific affinity for CB_2_. Other CB_2_ selective agonists were synthesized, such as GW405833 [138], which has anti-inflammatory properties [139,140,141]. However, GW405833 may also act as a non-competitive antagonist for CB_1_ as Li et al. noted in their study that the anti-allodynic effect of this compound was mediated through CB_1_ [142].

Further in vitro and in vivo studies revealed that targeting the CB_2_ receptor has immunomodulatory effects in several ways: the induction of apoptosis and anti-inflammatory cytokine production, as well as, the repression of cell proliferation, immune cell migration and pro-inflammatory cytokine production [6]. We list in Table 5 recent studies targeting CB_2_ in animal models of inflammation. Interestingly, in in vitro and in vivo studies in mouse models of ovalbumin-induced acute asthma, JWH-133 enhanced the mobilization of eosinophils [19]. Furthermore, in cecal-ligation- and puncture-induced polymicrobial sepsis in rats, JWH-133 induced an anti-inflammatory response by inhibiting apoptosis and NF-κB signaling in the brain, lung, liver and heart [143]. When administered to mice, HU308 reduced blood pressure, slowed defecation and caused anti-inflammatory effects [5]. HU910 was found to reduce the markers associated with liver injury in the ischemia/reperfusion injury mouse model and to attenuate the levels of pro-inflammatory cytokines and chemokines as well as immune cell infiltration [117].

Furthermore, in the context of mouse models of rheumatoid arthritis, JWH-133 induced an anti-inflammatory response by decreasing the production of proinflammatory cytokines and increasing the polarization of macrophages toward an M2 phenotype [144,145]. In addition, Fukuda et al. showed that, in collagen type-II antibody-induced arthritis, JWH-133 reduced arthritis scores and limited bone destruction [146]. Moreover, in the monoiodoacetic acid-induced osteoarthritis model, systemic and chronic administration of JWH-133 was associated with a reduction in pain-related behaviors [147]. In addition, experiments carried out in CB_2_-overexpressing mice suffering from monosodium iodoacetate-induced joint pain showed a better control of pain manifestation [148]. In the case of rheumatic diseases, CB_2_ agonists can improve inflammation and reduce pain concomitantly.

Taken together, these findings confirm that targeting CB_2_ with pharmacological agents can often improve inflammation in a wide range of inflammatory diseases, by modulating, directly or indirectly, the responses of various immune cells such as eosinophils [143], macrophages [149], neutrophils [54,56] or lymphocytes [15,150].

**Table 4:** CB_2_ selective ligands.

Compound	Function	CB_2_ Ki (nM)	CB_1_ Ki (nM)	References
JWH-133	CB_2_ Agonist	3.4	677	[137]
HU308	CB_2_ Agonist	22.7	10 μM	[5]
HU910	CB_2_ Agonist	6	1.37 μM	[117]
Gp1a	CB_2_ Agonist	0.037	363	[151]
JWH-015	Agonist	13.8	383	[13]
AM1241	Agonist	2	580	[152]
RO6871304	Agonist	17	10 μM	[153]
RO6871085	Agonist	76	-	[151]
GW405833	Agonist	12	4472	[138]
4Q3C	Agonist	8.5	10 μM	[154]
ABK-5	Agonist	28	-	[155]
S-777469	Agonist	36	4607	[156]
CP 55,940	CB_1_ and CB_2_ Agonist	0.68	0.58	[157]
WIN 55,212-2	CB_1_ and CB_2_ Agonist	3.30	1.9	[158]
SR145298	CB_2_ inverse agonist	0.6	10 μM	[135]
AM630	CB_2_ inverse agonist	31.2	5 μM	[159]

**Table 5:** Anti-inflammatory effects of CB_2_ activation by agonists in rodent models of inflammation.

Model	Species	Treatment	Dose/Route of Administration	Effects	References
Neuro-inflammation					
Brain ischemia	Mouse	JWH-133	1.5 mg/kg Intraperitoneal injection	↓ Microglia and macrophage infiltration ↓ CCL2, CCL3 and CCL5 ↓ IL-6, TNF-α ↓ iNOS	[160]
Hypoxic-ischemic encephalopathy			1.5 mg/kg Intraperitoneal injection	↑ Neuroprotection ↓ CCL2 ↓ TNF-α	[161]
Neuroinflammation in the rostral ventrolateral medulla			1 mmol/L in 10 μL Intracerebroventricular injection	↓ Blood pressure, heart rate ↓ Pro-inflammatory cytokines	[162]
Pentylenetetrazole-induced epilepsy					
Pentylenetetrazole-induced seizures			3 mg/kg Intraperitoneal injection	↓ Susceptibility to pentylenetetrazole-induced seizures	[163]
Postoperative cognitive dysfunction			3 mg/kg Intraperitoneal injection	↓ Surgery memory loss ↓ Pro-inflammatory cytokines	[164]
Stress-induced neuroinflammation			2 mg/kg Intraperitoneal injection	↓ CCL2 and TNF-α ↓ COX-2, iNOS and NF-κB	[165]
Subarachnoid hemorrhage			2 mg/kg Intraperitoneal injection	↓ Edema ↑ Expression of ZO-1 and blood–brain barrier integrity ↑ Expression of TGF-β1 ↓ Pro-inflammatory cytokines	[164]
L-dopa-induced dyskinesia (Parkinson’s disease model)		HU308	2 mg/kg Intraperitoneal injection	↓ Dyskinesia ↓ Microglia proliferation and cytokine release	[166]
Traumatic brain injury		HU910	5 to 10 mg/kg Intraperitoneal injection	↑ Neurobehavioral recovery ↑ Recovery of the cortical spinal tract ↓ TNF-α	[79]
		O-1966	5 mg/kg Intraperitoneal injection	↓ Microglia and macrophage infiltration ↓ Blood–brain barrier disruption	[167]
		Gp1a	1 to 5 mg/kg Intraperitoneal injection	↓ Edema and neurovascular injury ↑ Blood flow and improved neurobehavioral ↑ Macrophage polarization into M_2_ phenotype	[168]
Retrovirus-infection-induced neuropathic pain			5 mg/kg Intraperitoneal injection	↓ Allodynia ↔ Neuroinflammation	[169]
Germinal matrix hemorrhage-induced neuroinflammation	Rat	JWH-133	1 mg/kg Intraperitoneal injection	↑ Macrophage polarization into M_2_ phenotype ↓ Microglia accumulation ↑ Anti-inflammatory cytokines release ↓ TNF-α	[170] [171]
Intracerebral hemorrhage	Rat Mouse		1.5 mg/kg 1 mg/kg Intraperitoneal injection	↓ Edema ↑ Expression of ZO-1 and blood–brain barrier integrity ↑ Expression of TGF-β1 ↓ Pro-inflammatory cytokines	[164] [172]
Meningitis induced by *S.pneumonae*	Rat		1 mg/kg Intraperitoneal injection	↓ Microglia activation ↔ Hippocampal injury	[173]
Inflammation					
Cecal-ligation- and puncture-induced sepsis	Mouse	HU308	2.5 mg/kg Intraperitoneal injection	Protection against sepsis ↓ Activity of NLRP3 and Caspase-1 ↓ Cell pyroptosis	[125]
Corneal injury			0.5 to 1.5% *w*/*v* Topical application	↓ Neutrophil infiltration	[52]
Pneumonia-induced lung acute injury			3 mg/kg Intravenous injection	↑ IL-10 ↓ CXCL2 and TNF-α ↓ Acute lung injury score	[174]
LPS-induced interstitial cystitis			5 mg/kg Intraperitoneal injection	↓ Bladder inflammation	[175]
Sepsis			2.5 mg/kg Intravenous injection	↓ Adherent leukocyte in submucosal venules	[176]
LPS-induced interstitial cystitis		JWH-015	5 mg/kg Intraperitoneal injection	↓ Leukocyte infiltration ↓ Myeloperoxidase ↓ TNF-α, IL-1α and IL-1β	[177]
Trinitrobenzene sulfonic acid (TNBS)-induced colitis		AM1241	10 to 20 mg/kg Intraperitoneal injection	↓ Macroscopic damage and colitis ↓ Inflammation and MPO production	[178]
Rheumatoid arthritis		4Q3C	10 mg /kg Intraperitoneal injection	↓ Rheumatoid arthritis severity ↓ TNF-α and IL-1β	[144]
		JWH-133	1 mg /kg Intraperitoneal injection	↑ M2 polarization of macrophages ↓ TNF-α, IL1-β and IL-6 ↑ IL-10	[145]
Spinal cord injury		O-1966	5 mg/kg Intravenous injection	↓ Leukocyte infiltration ↓ CXCL9 and CXCL11 ↓ IL-23p19 and IL-23R ↓ TLR expression	[179]
Cecal-ligation- and puncture-induced sepsis (CLP) and sepsis	Rat	JWH-133	0.2 to 5 mg/kg Intravenous injection	↓ TNF-α, IL-1β, IL-6 ↑ IL-10	[143]
Intestinal ischemia/reperfusion		AM1241	0.1 to 10 mg/kg Intravenous injection	↓ TNF-α and IL-1β	[180]
Bile duct ligation			3mg/kg Intraperitoneal injection	↓ Apoptosis ↓ Pro-inflammatory cytokines ↑ IL-10	[181]
Endotoxin-induced uveitis		HU910 RO6871304 and RO6871085	1.5% *w*/*v* Topical application	↓ Eye inflammation ↓ Neutrophils migration	[153]
Carrageenan-induced paw oedema		GW405833	3 mg/kg Intravenous injection	↓ MPO activity ↓ Leukocyte recruitment	[140]
Concanavalin A-induced acute liver injury			20 mg/kg Intraperitoneal injection	↓ Liver damage and hepatocyte apoptosis ↓ Jurkat-T cell apoptosis	[139]
Acrolein-induced cystitis		GP1a	10 mg/kg Intraperitoneal injection	↓ Severity of cystitis ↓ Bladder inflammation	[182]
Pulmonary inflammation induced by *Mycobacterium bovis*			10 mg/kg Intraperitoneal injection	↓ Pulmonary inflammation ↓ Neutrophil accumulation ↓ CCL2, CXCL1, TNF-α and LTB_4_	[183]
Chronic ileitis model TNF ^RE/+^			5 mg/kg Retro-orbital injection	↓ Ileitis ↑ T-regulatory lymphocytes and IL-10	[184]
Incised skin wound model			3 mg/kg Intraperitoneal injection	↑ Keratinocyte migration and re-epithelialization ↓ Pro-inflammatory cytokines ↑ M_2_ macrophages	[185] [186]
Injection of complete Freund’s adjuvant (CFA) into the hind paw		ABK-5	5 to 20 mg/kg Intraperitoneal injection	↑ Jurka-T cell migration ↓ IL-2 and TNF-α ↓ CXCL12 chemotaxis	[187]
Allergy					
Antigen-induced dermatitis	Mouse	S-777469	10 to 30 mg/kg Oral administration	↓ Mast cell infiltration ↓ Eosinophil accumulation ↓ Block 2-AG activity ↓ Swelling	[188]
Intranasal-LPS inflammation	Mouse	URB597 JZL184	0.3 mg/kg Intraperitoneal injection −1 and 5 mg/kg intranasal administration	↓ Neutrophil in broncho alveolar ↓ TNF-α	[189]
Ovalbumin-induced asthma	Guinea pig	CP 55,940	0.1 mg/kg Intraperitoneal injection	↓ Myeloperoxidase ↓ Mast cell degranulation ↓ TNF-α and PGD_2_	[190]
Metabolic syndrome					
Diet-induced obesity	Mouse	JWH-133	5 to 10 mg/kg Intraperitoneal injection	↓ Weight gain ↑ Glucose tolerance and insulin sensitivity ↓ M_1_-associated markers and cytokine production	[191]
		HU308	4 mg/kg Intraperitoneal injection	↔ Weight gain ↓ M1 polarization of adipose tissue macrophages	[76]
Myocardial infarction		-JWH-133 -HU308	−1 to 10 mg/kg −2 mg/kg Intraperitoneal injection	↓ The severity of myocardial infraction and myocardial enzymes ↑ Myocardial viability ↓ NLRP3 activation ↓ Pro-inflammatory cytokines	[192]
Hepatorenal syndrome induced by bile duct ligation		HU910 10 mg/kg	10 mg/kg Intraperitoneal injection	↓ Liver and kidney fibrosis ↓ Inflammatory markers ↓ Oxidative damage	[193]
Inflammatory diabetic retinopathy			5 mg/kg Intraperitoneal injection	↓ Vascular permeability ↓ TNF-α, IL-1β, IL-6	[194]
Atherosclerosis		WIN 55,212-2	0.5 to 1 mg/kg Intraperitoneal injection	↓ Atherosclerotic lesion ↓ Macrophage infiltration ↓ CCL2, IL-6 and TNF-α	[195]
Myocardial ischemia-reperfusion injury			3.5 mg/kg Intraperitoneal injection	↓ Myeloperoxidase ↓ IL-1β and IL-8	[196]
Wistar Kyoto and spontaneous hypertensive line rats	Rat	JWH-133	1mmol/l Intracerebroventricular injection	↓ Blood pressure ↓ Pro-inflammatory cytokines	[162]

The arrows (↑/↓/↔) indicate whether a process is enhanced, reduced or unchanged respectively.

## 5. The Effect of CB_2_ Activation in Airway Inflammation

The recruitment of circulating immune cells is an amplification factor for pathologies with an inflammatory component [197]. This is the case in chronic allergic airway inflammation where the infiltration of the airways by eosinophils, mast cells and T helper lymphocytes is an important step of the pathogenesis [198]. Activation of both recruited and airway resident immune cells, namely innate lymphoid type 2 cells (ILC2), leads to the production of pro-inflammatory Th2 cytokines such as IL-4, IL-5 and IL-13 [198,199,200]. IL-4 production by Th2 lymphocytes also leads to the isotypic switch of B cells from IgM to IgE antibodies in the lungs [198]. These responses enhance mucus secretion by goblet cells, airway hyperresponsiveness and bronchoconstriction [201,202]. Since circulating immune cells express high levels of CB_2_ [17], their recruitment during an inflammatory pathology causes an influx of such receptors that have the potential to influence disease development.

Studies on healthy volunteers or asthmatic patients treated with Δ^9^-THC revealed a beneficial increase in bronchodilatation and specific airway conductance in treated individuals [203,204]. In the context of an ovalbumin-induced allergic reaction in mice, treatment with Δ^9^-THC attenuated the Th2 allergic response. Notably, it decreased the gene expression of IL-2, IL-4, IL-5 and IL-13, reduced serum IgE levels and diminished mucus secretion [205].

In order to understand the specific implication of CB_1_ and CB_2_ in the beneficial effects elicited by Δ^9^-THC, Newton and Klein studied the effect of this plant cannabinoid in a Th1-driven inflammatory reaction, i.e., that following *Legionella pneumophila* infection. They found that Δ^9^-THC decreased the serum levels of the Th1 cytokines, IL-12 and IFNγ by acting specifically through CB_1_. Δ^9^-THC also induced an increase in GATA3 signaling and IL-4 production by activating CB_2_ exclusively [123]. Furthermore, these authors demonstrated that, in ovalbumin-sensitized mice, CB_2_ activation by a selective agonist reduced IgE levels [206]. These results emphasize how, although Δ^9^-THC can tune down the inflammatory reaction of both Th1- and Th2-driven pathologies, this is the sum of independent effects mediated differently through CB_1_ and CB_2_ activation.

Some studies investigated the role of AEA and 2-AG in asthma. Zoerner et al. found that the bronchoalveolar fluids of allergic asthma patients challenged with an allergen contained much greater levels of AEA than patients given saline. Moreover, patients with higher AEA levels also had more severe symptoms of airway inflammation [207]. A study by Larose et al. showed that IL-3, IL-5 and GM-CSF greatly potentiate the CB_2_-dependant chemoattractant effect of 2-AG on eosinophils isolated from healthy donors [58]. In ovalbumin-sensitized guinea pigs, the inhibition of FAAH with URB597 reduced Th2 cytokine production and infiltration of immune cells but had marginal effects on airway hyperreactivity. Conversely, the inhibition of MAGL with JZL184 attenuated cytokine production, immune infiltration and airway hyperreactivity [107].

In a mouse model of ovalbumin-induced asthma, Frei et al. showed that the CB_2_ agonist JWH-133 increased the chemotaxis, activation and reactive oxygen species production of eosinophils. These effects were reduced in both *Cnr2*^−^*^/^*^−^ and eosinophil-deficient mice confirming that CB_2_ activation on eosinophils is a key event in allergic airway inflammation [19]. Furthermore, Hurrel et al. showed that CB_2_ signaling induced the proliferation and activation of ILC2 isolated from mouse lungs [208]. They confirmed, using *Cnr2*^−^*^/^*^−^ mice, that these animals presented reduced lung inflammation and airway hyperreactivity, which was a consequence of reduced ILC2 activation [208]. Ferrini et al. demonstrated that the absence of *Cnr2* in a model of common house dust mite sensitization increased NK cell infiltration and activation that prompted the secretion of Th1 cytokines in the airways. This activation of NK cells led to a drastically reduced number of ILC2 cells, which was accompanied by a reduction in Th2 cytokine levels [59].

The studies cited above, in addition to those we summarize in Table 6, suggest that the putative beneficial effect of CB_2_ activation is highly context-dependent. Nevertheless, we can emphasize that in a Th2 inflammatory context, including allergy, antagonism of CB_2_ is strongly beneficial since it prevents the inflammatory response possibly by switching it toward a Th1-type response.

**Table 6:** CB_2_ modulates the inflammatory response in vivo and in vitro.

Model	Treatment	Dose/Route of Administration	Effects	References
In vivo				
House dust mite inhalation (allergy)	*Cnr2* ^−/−^	-	↓ Th2 cytokine production ↑ NK cell number ↓ ILC2	[59,209]
Mice challenged with IL-33	*Cnr2* ^−/−^	-	↓ ILC2	[208]
Mice challenged with antigen/adjuvant OVA/Alum combination	*Cnr2* ^−/−^	-	↑ Serum level of IgE	[206]
Delayed-type hypersensitivity induced by methylated BSA	AEA or 2-AG	40 mg/kg Intraperitoneal injection	↑ IL-10 and Th2 cytokines ↓ Th1 and Th17 cytokines	[210,211]
*Legionella. pneumophilia* infection	Δ^9^-THC	4 mg/kg Intravenous injection	↓ Th1 cytokine production ↓ IFNγ production by splenocytes ↑ IL-4 production by splenocytes	[212]
Diet-induced obesity	JWH-133	5 to 10 mg/kg Intraperitoneal injection	↑ M2 macrophages polarization	[191]
In vitro				
Mouse-bone-marrow-derived macrophages	HU308	1 μM	↑ Macrophage polarization to M2	[213]
Mouse splenic B cells	SR144528	0.1 μM	↑ Class switch from IgM to IgE	[214]
Mouse dendritic cells	-Δ^9^-THC -2-AG	−5 μM −1 to 10 μM	↑ Apoptosis ↑ Th1 inflammatory response ↑ Dendritic cell migration	[215,216]
Human B cell line SKW 6.4	-SR144528 -AM630	−2.5 to 10 μM 0.63 to 2.5 μM	↓ IL-6-stimulated IgM secretion	[217]
Human T lymphocytes	SR144528	1 μM	↓ Th2 cytokine response	[218]
Primary-human-fibroblast-like synoviocyte osteoarthritis	HU308	1 μM	↓ CCL2, MMP1, MMP3 and IL-6	[213]
Human peripheral blood mononuclear cells	COR167	10^−3^ to 10 μM	Shift of Th1 phenotype toward Th2 phenotype ↓ Th17 ↓ IL-4 and IL-5 ↓ Chemokines	[219]
Human keratinocyte (HaCaT) cell	2 (1-adamantanylcarboxamido) thiophene derivatives	10 μM	↓ CCL2	[220]
Human bronchial epithelial cells (16HBE140^-^)	Virodhamine		↓ IL-8	[94]

The arrows (↑/↓) indicate whether a process is enhanced or reduced, respectively.

## 6. The Effect of Cannabinoid-Based Treatments in Inflammatory Diseases

**Figure 2:**
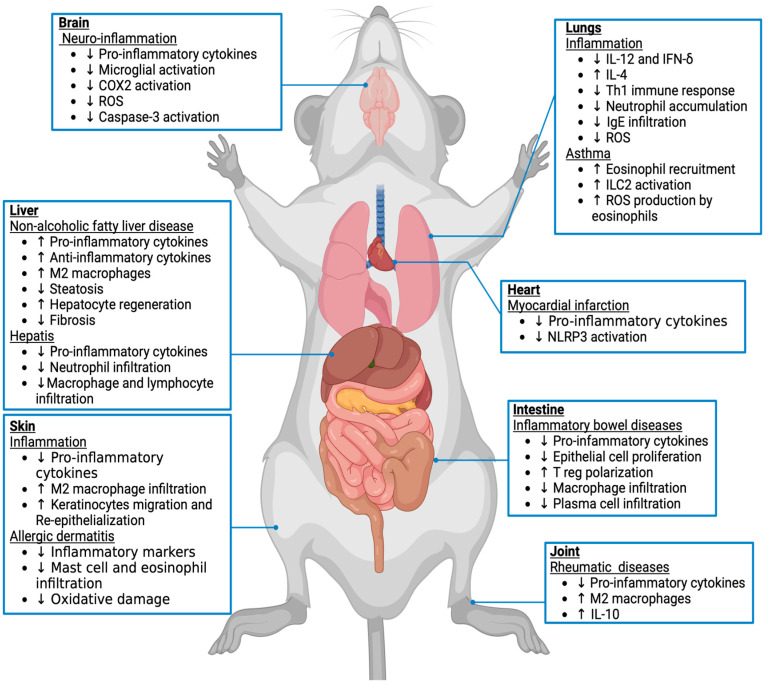
Effects of CB_2_ activation on inflammation in different mouse tissues and pathological condition. The endocannabinoid system via the CB_2_ receptor has been implicated in inflammatory processes. Based on previous studies in animal models, the arrows (↑/↓) indicate whether a process associated with each pathology is enhanced or reduced, respectively.

Tissue alterations in endocannabinoid concentrations and the expression of endocannabinoid receptors and metabolic enzymes have been associated with several inflammatory conditions, such as neuroinflammation and chronic inflammatory diseases (Figure 2). Based on accumulated findings, several clinical studies on cannabinoid therapies were instigated that we bring to light in Table 7.

**Table 7:** List of clinical trials based on endocannabinoids targeting inflammation.

Title	Compound	Phase	Intervention	Completed	Primary Outcomes	Outcomes Met	References
A study of The Effects Of CB_2_ Compound Of GW842166 In Patients With Osteoarthritis (NCT00479427)	GW842166 (CB_2_ agonist)	II	100 mg per os for 14 days.	YES	Change in pain scores from baseline to the end of treatment in Western Ontario and McMasters University Osteoarthritis Index (WOMAC) using the pain subscore for 6–8 weeks.	Unknown	-
Dental Pain 3rd Molar Tooth Extraction GW842166 (NCT00444769)	GW842166 (CB_2_ agonist)	IIa	Single dose of 100–800 mg per os, preoperative or postoperative.	YES	Decrease in Visual Analog Scale (VAS) pain intensity 10 h post-surgery.	NO	[221]
A Randomized, Double-Blind Study to Evaluate the Safety and Efficacy of 2 Doses of S-777469 in Patients With Atopic Dermatitis (NCT00703573)	S-777469 (CB_2_ agonist)	II	200–800 mg per os twice per day for 12 weeks.	YES	(1) Efficacy assessed by Physician’s Global Assessment (PGA) and Numerical Rating Scale (NRS). (2) Safety, determined by adverse event frequency and changes in laboratory values.	Unknown	-
A Phase Ib/IIa, Double-Blind, Randomized Study to Assess the Safety, Tolerability, Pharmacokinetics, and Pharmacodynamics of S-777469 in Subjects With Atopic Dermatitis (NCT00697710)	S-777469 (CB_2_ agonist)	Ib/IIa	50–800 mg per os twice per day for 1, 7 and 14 days.	YES	(1) Safety (adverse event monitoring, vital sign measurements, physical examination measurements, 12-lead electrocardiogram assessments and standard clinical laboratory safety tests (hematology, blood chemistry and urinalysis)). (2) Pharmacokinetic endpoints included Cmax, Tmax, T1/2,12hr, T1/2,z and AUC0-12h for each dose level of S-777469 based on the sampling schedule.	Unknown	-
Safety, tolerability, and efficacy of JBT-101 in subjects with dermatomyositis (NCT02466243)	JBT-101 (CB_2_ agonist)	II	20 mg per os daily for 28 days followed by 20 mg p.o. twice per day for 56 days.	YES	(1) Change in Cutaneous Dermatomyositis Disease Area and Severity Index (CDASI) from baseline. (2) Safety and tolerability measured by the number of participants with treatment-emergent adverse events.	YES	[222]
Safety, Tolerability, Pharmacokinetics, and Efficacy of JBT-101 (Lenabasum) in Cystic Fibrosis (NCT02465450)	JBT-101 (CB_2_ agonist)	II	20 mg per os twice per day for 5 to 12 weeks.	YES	Number of participants with treatment-emergent adverse events.	YES	[223]
Trial to Evaluate Efficacy and Safety of Lenabasum in Cystic Fibrosis (NCT03451045)	JBT-101 (CB_2_ agonist)	II	5 and 20 mg per os twice per day for 28 weeks.	YES	Rate of pulmonary exacerbation (PEx) over 28 weeks.	NO	[223] Available at: https://clinicaltrials.gov Reference: NCT03451045
Safety, tolerability, efficacy, and pharmacokinetics of JBT-101 in systemic sclerosis (NCT02465437)	JBT-101 (CB_2_ agonist)	II	5 and 20 mg per os twice per day for 28 days followed by 20 mg until day 84.	YES	(1) Number of participants with treatment-emergent adverse events from baseline at day 113. (2) Combined response index in diffuse Cutaneous Systemic Sclerosis (CRISS) at days 85 and 113.	YES	[224]
JBT-101 in Systemic Lupus Erythematosus (SLE) (NCT03093402)	JBT-101 (CB_2_ agonist)	II	5 or 10 or 20 mg per os daily for 12 weeks.	YES	Improvement in the maximum daily numeric rating scale for pain (NRS-Pain) score at day 84.	YES	[225]
Trial to Evaluate Efficacy and Safety of Lenabasum in Diffuse Cutaneous Systemic Sclerosis (RESOLVE-1) (NCT03398837)	JBT-101 (CB_2_ agonist) Hki=	III	5 or 20 mg per os daily for 52 weeks.	YES	Efficacy of Lenabasum compared to placebo for the American College of Rheumatology Combined Response Index in diffuse cutaneous Systemic Sclerosis score (ACR-CRISS).	NO	[226] [227]
Tolerability, Pharmacokinetics, and Efficacy of APD371 in Participants With Crohn’s Disease Experiencing Abdominal Pain (NCT03155945)	APD371 (CB_2_ agonist)	IIa	25 mg per os three times a day for 8 weeks.	YES	Number of participants with treatment-emergent adverse events (TEAEs) and serious adverse events (SAEs).	YES	[228]
Olorinab in Irritable Bowel Syndrome With Predominant Constipation (IBS-C) and Irritable Bowel Syndrome With Predominant Diarrhea (IBS-D) (CAPTIVATE) (NCT04043455)	APD371 (CB_2_ agonist)	II	10, 20 or 50 mg per os three times a day for 12 weeks.	YES	Change in patient-reported average abdominal pain score (AAPS) from baseline to week 12.	NO	[229]
Effect of Olorinab on Gastrointestinal Transit in Patients With Irritable Bowel Syndrome (NCT04655599)	APD371 (CB_2_ agonist)	Ib	Olorinab per os, three times a day for 4 days with a final dose on day 5.	NO	(1) Colonic transit geometric center after consumption of radiolabeled meal, based on the delivery of activated charcoal in a methacrylate-coated capsule. (2) Gastric emptying half-life (t½) as determined by scintigraphic imaging of radiolabeled meal.	Terminated	-
A Study of LY2828360 in Patients With Osteoarthritic Knee Pain (NCT01319929)	LY2828360 (CB_2_ agonist)	II	80 mg per os LY2828360 for 4 weeks.	YES	Change from baseline to 4-week endpoint in weekly mean of daily 24 h Average Pain Scores (APS).	NO	[230]
Study to Investigate the Analgesic Efficacy of a Single Dose of AZD1940 (NCT00659490)	AZD1940 (dual CB_1_/CB_2_ agonist)	II	Single dose per os of 100 or 800 μg.	YES	Pain Area Under the Curve 0–8 h (AUC0-8h).	NO	[231]
Study to Investigate the Safety, Tolerability and Pharmacokinetics of AZD1940 (NCT00689780)	AZD1940 (dual CB_1_/CB_2_ agonist)	I	Single dose per os of 100 or 800 μg.	YES	Assessment of adverse events (AEs) occurring during the study, blood pressure (supine and standing), pulse rate, respiratory rate, body temperature, laboratory variables and ECG.	NO	[231]
TT-816 as Monotherapy or in Combination With a PD-1 Inhibitor in Patients With Advanced Cancers (SEABEAM) (MK3475-E88) (NCT05525455)	TT-816 (CB_2_ antagonist)	I and II	-	NO	Incidence of adverse events (AEs) and serious adverse events (SAEs) (Phase 1). Incidence and nature of dose-limiting toxicities (DLTs) (Phase 1). The Maximum Tolerated Dose (MTD) or Recommended Phase 2 Dose (RP2D) of oral TT-816 as monotherapy (Phase 1m). The Maximum Tolerated Dose (MTD) or Recommended Phase 2 Dose (RP2D) of oral TT-816 in combination with a PD-1 inhibitor (Phase 1p). Overall Response Rate. Scale: confirmed Complete response (CR) or Partial response (PR), Duration of Response (DOR) and Disease Control Rate (DCR). Changes from baseline in clinical safety laboratory values and vital signs.	-	[232]

Source: clinicaltrials.gov accessed on mars 2024.

### 6.1. Potential in Neuroinflammation: Alzheimer’s Disease

Mentioned for the first time in 1995 [233], neuroinflammation is characterized by an inflammatory response within the brain or spinal cord and reported as an indicator and modulator of neurodegeneration [234]. Several studies have highlighted the importance of the CB_2_ receptor in diseases associated with neuroinflammation [235,236]. The CB_2_ receptor is expressed by several cells in the brain such as astrocytes, reactive microglia, perivascular microglia, oligodendrocytes, neuronal progenitor cells and cells involved in blood–brain barrier integrity [237]. In 2003, Benito et al. found that the *CNR2* gene was highly expressed in post mortem brain microglia from subjects with Alzheimer’s disease, whereas the expression of the *CNR1* gene remained unchanged [238]. In vitro studies demonstrated that CB_2_ activation by JWH-015 suppressed the production of IFN-γ, TNF-α and nitrous oxide by microglia after stimulation with the amyloid β peptide [239]. In addition, JWH-133 blocked the activation of microglia by amyloid β peptide, and this treatment with the CB_2_ agonist reduced the production of pro-inflammatory cytokines [240]. Furthermore, when neurons and microglia were co-cultured, treatment with JWH-133 prevented the microglia-mediated toxicity of neurons after amyloid β peptide exposure [241]. These in vitro results suggest that the beneficial effects of CB_2_ activation are associated with the presence of this cannabinoid receptor on microglia and are related to the suppression of their pro-inflammatory activation.

Interestingly, the levels of AEA were found to be lower in the frontal and temporal cortex tissues of post mortem patients with Alzheimer’s disease compared to control subjects [242]. Furthermore, higher 2-AG plasma levels correlated with better memory and attention in patients with Alzheimer’s disease. This suggests a protective mechanism associated with the modulation of the endocannabinoid system [243].

Since it is possible to modulate endocannabinoid levels by inhibiting their hydrolysis, JZL184, which is an irreversible inhibitor of MAGL, was used in an Alzheimer’s disease mouse model. Pihlaja et al. observed a decrease in the production of pro-inflammatory mediators by microglia and astrocytes isolated from adult mice treated with JZL184 [244]. The inhibition of MAGL has been associated with several positive effects on Alzheimer’s disease in animal models, such as amelioration of inflammation and neuronal disorders [245]. The inhibition of FAAH activity with a selective irreversible inhibitor, UBR597, increased the availability of AEA [246]. Chiurchiu et al. demonstrated that macrophages from patients with Alzheimer’s disease treated with URB597 produced less pro-inflammatory cytokines [247]. Moreover, the treatment of aged rats with URB597 reduced the expression of IL-1β and TNF-α and restored aged-related disorders [248].

In a transgenic Tg APP 2576 mouse model of Alzheimer’s disease that overexpresses the amyloid precursor protein, the authors observed that chronic treatment with JWH-133 was able to decrease microglia activation and reduce COX-2 activation and TNF-α production. More importantly, CB_2_ activation helped to reduce cognitive impairment [241]. In the APP/PSI mouse model expressing a chimeric human/mouse amyloid precursor protein directly in neurons, the administration of JWH-133 was found to be effective at reducing Tau-hyperphosphorylation [249].

These results support the idea that targeting CB_2_ and endocannabinoid metabolism is a therapeutic option for some neuroinflammatory diseases.

### 6.2. Potential in Chronic Inflammation: Inflammatory Bowel Diseases (IBDs)

Crohn’s disease and ulcerative colitis are the two major chronic idiopathic IBDs [250]. Though several clinical and pathological features differ between these two IBDs [251], both diseases are characterized by intestinal inflammation and alteration of the epithelial barrier [252]. This leads to the translocation of bacteria and microbial products from the gut lumen through the intestinal wall. Consequently, an acute inflammatory reaction occurs, which is driven by immune cell infiltration and cytokine production [253]. As the disease progresses, an increasingly uncontrolled chronic inflammation develops that leads to tissue destruction [254,255].

Two noteworthy independent studies on IBD patients compared users versus nonusers of cannabis. Storr et al. showed that patients who used cannabis for more than six months were more susceptible to undergoing surgical treatment associated with Crohn’s disease [256]. On the other hand, Mbachi et al. compared two groups of patients with Crohn’s disease, one group of non-cannabis users and a second group of cannabis users. They concluded that cannabis users developed fewer complications, such as fistulizing disease, colectomy or intra-abdominal abscess [257]. Furthermore, mucosal tissue from the inflamed region of the colon of IBD patients incubated with the CB_2_ agonist JWH-133 for 6 h showed increased epithelial cell proliferation accompanied by decreased MMP-9 and IL-8 secretion [258]. This suggests that, in the context of IBD, the protective effect induced by plant cannabinoids might be mediated by the CB_2_ receptor.

Even though CB_2_ is highly expressed by infiltrating macrophages and plasma cells [23], the receptor is also detected in the esophagus, stomach, ileum and intestine [259]. It is expressed by epithelial cells, goblet cells and Paneth cells and increased during the acute phase of IBDs [260]. In addition, the Q63R functional variant of the CB_2_ protein has recently been significantly associated with Crohn’s disease and ulcerative colitis [261].

It is demonstrated that plasma AEA levels are higher in patients with IBD [262]. Regarding 2-AG, its levels are higher in patients with Crohn’s disease and are associated with increased expression of the 2-AG synthesizing enzyme DAGL-α [263].

Several IBD mouse models such as 2,4,6-Trinitrobenzenesulfonic acid- or Dinitrobenzene sulfonic acid-treated mice are used to mimic colitis [264]. First, in *Tnfα* overexpressing mice treated with TNBS/DSS, it was demonstrated that expression of *Cnr2* was increased in immune cells. Furthermore, GP-1a treatment induced the polarization of T lymphocytes into regulatory phenotype secreting IL-10 [184]. In the DNBS-induced IBD model, JTE907, a CB_2_-specific inverse agonist, induced phenotypic differentiation of inflammatory T cells into *foxp3* positive regulatory T cells secreting TGF-β and IL-10. In these studies, a CB_2_ receptor agonist reduced the severity of the disease [265].

In mice with DSS-induced IBD, treatment with *N*-arachidonoyl-serotonin, an inhibitor of FAAH, helped to improve clinical scores and pathogenesis [262]. FAAH blockade decreased the number of macrophages, neutrophils, NK and NKT cells in the Peyer’s patches and colonic lamina propria. This treatment reduced systemic and colonic inflammatory cytokine levels [266]. The FAAH inhibitor PF-3845 ameliorated TNBS-induced colitis [267]. Moreover, URB597, another FAAH inhibitor, attenuated TNBS-induced colitis, and this anti-inflammatory effect was abolished when *Cnr1* and *Cnr2* were genetically deficient [268]. Increasing the availability of 2-AG by using the MAGL selective inhibitor JZL184 attenuated TNBS-induced murine colitis [269].

These results suggest that targeting CB_2_ and manipulating pharmacologically the availability of endocannabinoids are potential therapeutic avenues to improve the quality of life of patients with IBD in terms of pain and disease symptoms but also to improve inflammation associated with these disorders.

### 6.3. Potential in Metabolic Disease: Non-Alcoholic Fatty Liver Disease (NAFLD)

NAFLD encompasses a number of liver diseases ranging from isolated hepatic steatosis to steatohepatitis and irreversible cirrhosis [270]. NAFLD reflects the inability of the adipose tissue to perform its function as fat-storage tissue, leading to increased triglyceride uptake by hepatocytes [271]. The prevalence of NAFLD increases dramatically with obesity [272], dyslipidemia and type 2 diabetes [273] and is becoming the most common liver disease in developed countries [274]. Steatosis without any signs of fibrosis is considered an early condition, whereas the presence of fibrosis predicts chronic progression to severe liver disease [275].

A study with hepatitis-C-positive patients revealed that cannabis users had decreased prevalence of liver cirrhosis, although this did not improve mortality [276]. Furthermore, a study comparing the evolution of liver diseases in obese patients suggested that cannabis reduced the prevalence and progression of steatohepatitis [277].

It was established that *Cnr2* mRNA is expressed in the liver of morbidly obese women at different stages of NAFLD. Its expression was correlated with the expression of anti-inflammatory and pro-inflammatory mediators, indicating that CB_2_ may have a dual role in NAFLD and NAFLD-related complications [278]. Indeed, because of the high expression of *Cnr2* in the damaged liver, it was proposed that this phenomenon could predict the progression of liver disease from chronic hepatitis to irreversible cirrhosis and hepatocellular carcinoma [279]. *Cnr2* was expressed by hepatocytes but only in patients with non-alcoholic steatosis [280]. In addition, *Cnr2* was also expressed by Kupffer cells which are key players in immune control in the liver. Kupffer cells recognize many pathogens through various pattern recognition receptors and respond by producing pro-inflammatory cytokines [281]. Furthermore, the depletion of Kupffer cells in rats fed with a high-fat diet to induce steatosis protects against hepatic steatosis and insulin resistance, highlighting the importance of these cell types in liver disease [282]. These cells are very plastic in response to their environment as they can switch from a pro-inflammatory M_1_ to an anti-inflammatory M_2_ phenotype [283]. Kupffer cells isolated from *Cnr2* knock-out mice are more polarized toward an M1 phenotype compared to Kupffer cells form wild-type mice [284]. The absence of *Cnr2*, or treatment with the CB_2_ antagonist, AM630, also confers to mice protection against steatosis induced with a high-fat diet [55]. On the other hand, rats treated with the agonist AM1241 displayed significant expression of hepatic progenitor cell markers, which indicates that stimulating CB_2_ enhances hepatocyte regeneration [181]. Furthermore, chronic activation of CB_2_ with JWH-133 in cirrhotic rats decreased the arterial pressure, immune cell infiltration and the number of activated stellate cells but more importantly, decreased fibrosis [285].

Taken together, these findings from various human studies and mouse models reveal contradictions in the effects of AEA and 2-AG in liver injury and cast doubt on the value of CB_2_ as a therapeutic target for liver diseases. Further studies are warranted to confirm whether (1) CB_2_ has a beneficial role in NAFLD and (2) the anti-inflammatory effect of CB_2_ is enough to counterbalance its proposed effect of increasing lipid accumulation. It is perhaps more interesting to explore the possibility that endocannabinoids and CB_2_ expression are biomarkers of the evolution of liver disease.

## 7. Conclusions and Future Direction

In recent years, we have witnessed an upsurge in research aimed at better understanding the role of CB_2_ in different inflammatory contexts. Many animal models demonstrate that CB_2_ stimulation by endogenous and exogenous ligands leads to an anti-inflammatory response and improves the symptoms of inflammatory diseases. However, it is important to consider the inflammatory context in which the CB_2_ receptor is targeted. This is demonstrated in allergic airway inflammation, a Th2-driven response, for which CB_2_ activation is detrimental.

Specific agonists and antagonists were developed and used in several clinical studies to target the endocannabinoid system in inflammatory conditions (Table 6). Although only a fraction of these studies have reached successfully their primary outcome, it establishes CB_2_ as a promising therapeutic target. Therefore, future efforts should focus on developing CB_2_ ligands activating specific signaling pathways and establishing which ligands are effective in the inflammatory context of each disease.
